# Molecular Mechanisms Regulating Cytotoxic Lymphocyte Development and Function, and Their Associations to Human Diseases

**DOI:** 10.3389/fimmu.2014.00279

**Published:** 2014-06-11

**Authors:** Konrad Krzewski, Yenan T. Bryceson

**Affiliations:** ^1^Receptor Cell Biology Section, Laboratory of Immunogenetics, National Institute of Allergy and Infectious Diseases, National Institutes of Health, Rockville, MD, USA; ^2^Center for Infectious Medicine, Department of Medicine, Karolinska Institutet, Karolinska University Hospital Huddinge, Stockholm, Sweden

**Keywords:** cytotoxicity, lytic granules, secretory lysosomes, perforin, granzyme, immunological synapse, hemophagocytic histiocytosis, immune therapy

Cytotoxic lymphocytes, encompassing cytotoxic T cells and natural killer (NK) cells, play a pivotal role in immune defense. By directed release of perforin-containing lytic granules, cytotoxic lymphocytes can eradicate pathogen-infected, tumorigenic, and otherwise stressed cells. By the virtue of cytokine and chemokine secretion, they can also influence other cells of the immune system. In addition, cytotoxic lymphocytes can kill activated immune cells to limit excessive immune reactions and maintain homeostasis. In recent years, much progress has been made with respect to the mechanisms by which cytotoxic lymphocytes develop, differentiate, and exert their effector functions. In this Research Topic, we collected several exciting articles that highlight the mechanisms controlling the development and effector function of cytotoxic lymphocytes, as well as the role these cells play in several disease conditions.

We open the Research Topic with a review providing an up-to-date view on the development and differentiation of NK cells, and their relationships to other innate lymphoid cells. The authors describe in detail NK cell developmental progression through distinct stages before reaching the mature phenotype, and discuss possible reasons behind functional and phenotypic plasticity among NK cells from different tissues (1). With respect to differentiation, NK cells receive cues from several other cell types. Of particular interest is the ability of NK cells and dendritic cells to influence each other’s function during early immune responses. Such reciprocal interactions are the focus of a fascinating review by Chijioke and Munz (2).

Next, we take a closer look at series of molecular events shaping the response of cytotoxic lymphocytes. A research article by Olofsson et al. (3) demonstrates how stimulation by IL-2 influences the migration of individual NK cells and their recognition and killing of target cells. Then, a review by Galandrini et al. (4) discusses the mechanisms underlying activation of cytolytic machinery following target cell recognition. Two complementary reviews focus on the maturation and release of lytic granules, and the high-resolution techniques that allow for visualization of lytic granules and quantification of their subsequent fusion with the plasma membrane at the contact site with target cells (5, 6). The following research article shows how inhibitory signals interrupt the formation of activating synapses formed by primary NK cells, thus disrupting NK cell cytotoxicity (7). Finally, Baginska et al. (8) present a fascinating view from the opposite, target cell side, providing us with a comprehensive review of how tumor cells can affect the function of NK cells and evade cell death; these include secretion of multiple tumor-derived immunosuppressive factors and metabolites, or autophagy-mediated resistance to NK-mediated killing.

In a clinical perspective, a wide variety of mutations impairing cytotoxic lymphocyte development and/or function have been associated with severe diseases in humans. Impaired activity of cytotoxic lymphocytes has been linked to an increased susceptibility to viral infections, persistent inflammation, cancer, and autoimmunity. In the middle part of our Research Topic, we gathered articles that describe defects in cytotoxic lymphocyte biology and their relation to different human disease conditions. Focusing on NK cells, Ham and Billadeau (9) summarize a variety of human immunodeficiency syndromes that affect lymphocyte cytotoxicity, bridging the molecular, and clinical side of this Research Topic. A Hypothesis and Theory article introduces the interesting concept of “perforinopathy” (10). The authors discuss how immune dysregulation and immunopathology are caused by, or related to decreased perforin activity and/or delivery to the target cells, and present compelling evidence that this may be more common cause of human disease than previously assumed. Sieni et al. (11) provide an extensive view of hemophagocytic lymphohistiocytosis, an immunodeficiency caused by defective lymphocyte cytotoxicity, and describe how the knowledge gained by characterization of patients with hemophagocytic syndromes contributes to our understanding of cytotoxic lymphocyte function. This in-depth review is followed by two outstanding papers that illustrate the effect of mutations affecting cytotoxic lymphocyte activity in hemophagocytic lymphohistiocytosis. Jessen et al. (12) compare different molecular defects in lytic granule-associated lytic pathway with the clinical phenotype of hemophagocytic lymphohistiocytosis; based on the degree of impairment of cytotoxic lymphocyte activity a gradient of disease manifestations is distinguished, both in human hemophagocytic lymphohistiocytosis and the mouse models of the disorders. Muller et al. (13) characterize a novel missense mutation in syntaxin-11, and demonstrate that this mutation abrogates binding between syntaxin-11 and Munc18-2, an interaction critical for release of lytic granules from cytotoxic lymphocytes. Thus, the authors identify an important mechanism that could underlay the development of the familial hemophagocytic lymphohistiocytosis. The last article in this part of the Research Topic focuses on CD57 molecule in NK cell function, its expression pattern during NK cell differentiation and, importantly, in several pathologies, such as cancer, autoimmunity, and infections. The review compiles our current knowledge about the role of CD57 as a marker for NK cell function and disease association (14).

Lymphocyte cytotoxic activity may be harnessed therapeutically to target tumor cells in different adoptive cellular therapy regimes or through the use of recombinant antibodies. In this regard, Mentlik James et al. (15) describe and discuss different therapies available to enhance NK cell anti-tumor responses, with a particular focus on the use of a variety of therapeutic antibodies and immuno-modulating drugs to fight hematologic malignancies. Della Chiesa et al. (16) review the response of NK cells to human cytomegalovirus. The authors summarize evidence for how cytomegalovirus infection can promote NK cell development and maturation, which could actually be beneficial for the hematopoietic stem cell transplant therapies, due to accelerated anti-leukemic or even anti-viral responses. In addition, the aforementioned review by Chijioke and Munz (2) highlights the potential therapeutic benefits of dendritic cell–NK cell interactions, from enhancing anti-tumor NK cell responses, to dendritic cell-mediated expansion of tumor-specific T cells, to decreasing graft-versus-host-disease in different transplantation settings.

We hope that the reader will find this Research Topic very interesting and informative. We invite you to read the following articles and immerse yourself in the fascinating world of cytotoxic lymphocytes and their function.

## Conflict of Interest Statement

The authors declare that the research was conducted in the absence of any commercial or financial relationships that could be construed as a potential conflict of interest.
